# Relapse of Steroid‐Dependent Nephrotic Syndrome Despite Long‐Term Ciclosporin Therapy in an 11‐Year‐Old Child: A Case Report and Review on the Management of Nephrotic Syndrome in Children

**DOI:** 10.1155/crin/1396986

**Published:** 2026-08-16

**Authors:** Abdullah Faiz Zaihan, Nurdiana Jamil, Chia Siang Kow

**Affiliations:** ^1^ Department of Pharmacy, Shah Alam Hospital, Shah Alam, Selangor, Malaysia; ^2^ Faculty of Pharmacy, University of Cyberjaya, Cyberjaya, Selangor, Malaysia; ^3^ School of Pharmacy, IMU University, Kuala Lumpur, Malaysia, imu.edu.my; ^4^ School of Applied Sciences, University of Huddersfield, Huddersfield, UK, hud.ac.uk

## Abstract

**Background:**

Frequently relapsing nephrotic syndrome (FRNS) and steroid‐dependent nephrotic syndrome (SDNS) are distinct phenotypes defined by relapse frequency and the temporal relationship of relapse to prednisolone therapy, respectively. Both remain therapeutic challenges despite the use of corticosteroid‐sparing agents. Relapses may be triggered by intercurrent infections and can be complicated by significant edema, hypoalbuminemia, and infection risk, requiring prompt optimization of immunosuppressive and supportive therapy.

**Case Presentation:**

An 11‐year‐old boy with SDNS presented with a two‐day history of bilateral periorbital swelling and facial puffiness following fever, rhinorrhea, and productive cough. He had experienced 11 previous relapses and was receiving ciclosporin 50 mg twice daily and enalapril 5 mg once daily with good adherence. Previous kidney biopsy showed minor glomerular change, and ciclosporin trough concentration was therapeutic. On admission, he was febrile (38.3°C) with leukocytosis, neutrophilia, thrombocytosis, marked hypoalbuminemia, significant proteinuria, and hematuria. Penicillin V was initiated for spontaneous bacterial peritonitis prophylaxis. Prednisolone was optimized from 40 mg once daily to 30 mg twice daily based on a body surface area of 1.17 m^2^. Progressive edema and weight gain required escalation to intravenous frusemide with 20% human albumin, resulting in marked clinical improvement. Proteinuria, hematuria, and ascites resolved, and he was discharged clinically stable with minimal residual edema.

**Conclusion:**

This case highlights several important therapeutic considerations in relapsing childhood nephrotic syndrome: recognition of SDNS as a subgroup of SSNS, accurate prednisolone dosing during relapse, careful assessment of edema and intravascular volume status before diuretic therapy, and individualized use of steroid‐sparing agents and antimicrobial prophylaxis. In children receiving prolonged ciclosporin therapy, treatment should be regularly reviewed with blood pressure, renal function, and therapeutic drug monitoring in view of potential calcineurin inhibitor toxicity, and alternative steroid‐sparing options such as levamisole may be considered where clinically appropriate.

## 1. Introduction

Childhood nephrotic syndrome is one of the most common kidney diseases found among children. It is a disease characterized by hypoalbuminemia (< 25 g/L), proteinuria of > 40 mg/m^2^/hour (> 1 g/m^2^/day), edema, and hypercholesterolemia [1]. Childhood nephrotic syndrome is rare, with an average worldwide incidence varying from 2 to 16.9 cases per 100,000 children [2]. Approximately 90% of these cases are idiopathic or of unknown cause.

In nephrotic syndrome, defects of the glomerular resulted in leakage of high‐molecular‐mass proteins. As a result, many important hemostatic proteins of similar sizes are lost in the urine. Loss of albumin in the urine leads to a decrease in oncotic pressure, causing the fluid to shift from intravascular to the interstitial compartment. Decreased or expanded effective circulatory volume or both may result in the formation of edema. On the other hand, loss of other high molecular mass proteins such as protein S, antithrombin, fibrinogen, and Factors V and VIII will lead to hypercoagulability state, which explains the higher risk of venous thromboembolism among those with nephrotic syndrome [3, 4]. Another complication often associated with nephrotic syndrome is hyperlipidemia. This occurs as a result of abnormal glomerular permeability to plasma protein, hence increasing the hepatic synthesis of lipids and apolipoproteins as well as the reduction in clearance of chylomicrons and very low‐density lipoproteins [5, 6].

The first‐line therapy for the first episode of childhood nephrotic syndrome is oral corticosteroids. Around 80% of children with childhood nephrotic syndrome respond to oral corticosteroid therapy and achieve remission; these patients are classified as having steroid‐sensitive nephrotic syndrome (SSNS). Although children with SSNS respond to corticosteroids, many subsequently experience relapses, and some develop a frequently relapsing or steroid‐dependent disease course. Frequently relapsing nephrotic syndrome (FRNS) and steroid‐dependent nephrotic syndrome (SDNS) are therefore distinct relapsing patterns within SSNS. FRNS is defined according to the number and timing of relapses, whereas SDNS is characterized by consecutive relapses occurring during the recommended prednisolone therapy or within 14 days after its discontinuation [7]. Unfortunately, 75%–80% of children with SSNS will experience one or more relapses, with approximately 50% developing frequent relapses or steroid dependence. Children with FRNS or SDNS are at higher risk of steroid‐induced adverse effects such as obesity, growth impairment, hypertension, diabetes mellitus, osteoporosis, and adrenal insufficiency. Those who do not respond to corticosteroid therapy are classified as having steroid‐resistant nephrotic syndrome (SRNS), and this group is at higher risk of progression to renal failure [8–10]. Although effective treatment is available, progression toward renal failure and early mortality may occur if the disease is left untreated, especially among children with complicated relapsing disease and SRNS [11]. For children with FRNS, SDNS, or steroid toxicity, corticosteroid‐sparing treatment with cyclophosphamide, ciclosporin, mycophenolate mofetil, levamisole, or rituximab may be considered [1, 7, 8, 10, 12]. This case is reported in accordance with the CARE guideline [13] and focuses on the management of a child with SDNS, including glucocorticoid dosing, ciclosporin monitoring, edema management, and infection‐related treatment decisions.

## 2. Case

An 11‐year‐old boy was brought to the emergency department of a secondary hospital by his parents due to the sudden onset of bilateral periorbital swelling and facial puffiness for the past two days. Further history taking through his mother revealed that the patient had fever, runny nose, and chesty cough for the past three to four days, and his protein urine dipstick test was 3+ one day before the current encounter.

The patient had SDNS, a subgroup of SSNS. The patient was previously treated with oral cyclophosphamide; however, because he experienced disease relapse during cyclophosphamide therapy in the setting of steroid‐related toxicity, the treating pediatric nephrologist changed his glucocorticoid‐sparing therapy to ciclosporin, which he had subsequently received for five years. He had a total of 11 episodes of relapse since he was first diagnosed with nephrotic syndrome, and his most recent episode of relapse was one year ago. He is currently receiving oral ciclosporin 50 mg BD (100 mg/day), equivalent to approximately 2.8 mg/kg/day using his lowest recorded weight of 35.9 kg, as glucocorticoid‐sparing therapy and enalapril 5 mg OD as antiproteinuric therapy. Kidney biopsy performed in January 2021 showed minor glomerular change, a minimal‐change pattern compatible with his steroid‐sensitive phenotype. Compliance assessment was performed, and he was found to have good adherence to his medications, as his parents supervised the administration and consumption of his medications.

Upon presentation, his vital signs were all normal except that he had a temperature spike of 38.3°C. Blood investigations revealed neutrophilic leukocytosis (white blood cell count: 17.8 × 10^9^/L; normal: 4–11 × 10^9^/L and absolute neutrophil count: 13.3 × 10^9^/L; normal: 2–7 × 10^9^/L), consistent with an intercurrent infection but not sufficient alone to establish a bacterial etiology, and thrombocytosis (platelet: 529 × 10^9^/L; normal: 150–400 × 10^9^/L) in the context of nephrotic syndrome and inflammation. The preceding fever, rhinorrhea, and productive cough were considered a probable respiratory trigger for relapse; no microbiologically confirmed pathogen was reported. Liver function tests revealed that he had marked hypoalbuminemia (albumin: 8 g/L; normal: 35–50 g/L) due to nephrotic syndrome. He had fluctuations in his daily body weight due to noncompliance with fluid restrictions in addition to the use of diuretics and human albumin for diuresis. He was kept on negative fluid balance throughout his stay. His urine protein and blood level were markedly elevated (4+ and 2+, respectively) on admission but improved and normalized throughout his stay.

Upon admission, he was immediately initiated on oral penicillin V 250 mg BD according to local practice for the prophylaxis of spontaneous bacterial peritonitis, although routine prophylaxis in childhood nephrotic syndrome is not universally recommended. The antibiotic therapy was escalated to oral penicillin V 250 mg QID on Day 3 of admission in response to raised white blood cell and neutrophil counts compared to baseline, representing empirical treatment‐intensity dosing despite the absence of a confirmed bacterial focus.

In addition to the antibiotic therapy, oral ciclosporin 50 mg BD and enalapril 5 mg OD were continued throughout his stay. Continuation of ciclosporin was considered reasonable because he remained hemodynamically stable, tolerated oral medication, and had no documented severe sepsis, acute kidney injury, or calcineurin‐inhibitor toxicity. Oral prednisolone 40 mg OD was prescribed on Day 1 of admission for treatment of the nephrotic syndrome relapse. However, because this was below the recommended relapse dose, the regimen was revised on Day 3 to oral prednisolone 30 mg twice daily (total 60 mg/day). Using his height of 138 cm and lowest recorded weight of 35.9 kg, his BSA was 1.17 m^2^ by the Mosteller formula. The BSA‐based dose of 60 mg/m^2^/day would calculate to approximately 70.4 mg/day, while the weight‐based dose of 2 mg/kg/day would calculate to 71.8 mg/day. Both calculations exceeded the recommended maximum of 60 mg/day; therefore, the revised total dose of 60 mg/day was the correct capped relapse dose and was not underdosing. Separate stress‐dose hydrocortisone was not required because prednisolone 60 mg/day was already substantially above usual stress‐dose glucocorticoid equivalence, and he had no hemodynamic instability, persistent vomiting, diarrhea, or inability to absorb oral medication [14]. Therapeutic drug monitoring was performed, and the ciclosporin trough concentration was 128 μg/L (equivalent to 128 ng/mL), within the local laboratory range of 125–250 μg/L and the KDIGO 12 h trough range of 60–150 ng/mL [1]. IPNA recommends a narrower trough range of 60–100 ng/mL, emphasizing use of the lowest concentration that maintains remission [7].

As the patient presented with symptoms of fluid overload due to nephrotic syndrome, diuretic therapy was also given. Due to the risk of hypovolemia, diuretic therapy was not started until Day 4 of admission when he developed pitting edema, worsening facial and periorbital edema, as well as lung congestion. Serum electrolytes and renal function were reviewed before diuretic therapy. Although serum potassium had previously been mildly elevated at 5.4 mmol/L, it had decreased to 4.2 mmol/L before oral spironolactone 25 mg BD was initiated. Due to the marked increase in body weight and worsening edema on Day 6 of admission, diuretic therapy was escalated with intravenous (IV) human albumin 20% 10 g in combination with IV frusemide 30 mg, with clinical monitoring for intravascular depletion and electrolyte disturbance.

The patient was treated for a total of nine days in the pediatric ward. Signs and symptoms associated with relapsing nephrotic syndrome improved significantly following the treatment given. Proteinuria, hematuria, and ascites improved significantly. Upon discharge, he had only minimal pedal, facial, and periorbital edema. He was discharged with oral penicillin V 250 mg BD, oral prednisolone 30 mg twice daily (total 60 mg/day) until complete remission, defined as negative or trace urine protein for at least three consecutive days, followed by 40 mg as a single dose on alternate mornings for four weeks without tapering, oral ciclosporin 50 mg BD, and oral enalapril 5 mg OD.

### 2.1. Management of Relapsing Nephrotic Syndrome–Immunotherapy

Nephrotic syndrome is often associated with complications such as edema, hypertension, risk of infection, hyperlipidemia, and venous thromboembolism. The immediate goal of therapy in the management of nephrotic syndrome is to induce remission as soon as possible and to prevent immediate complications such as venous thromboembolism, infection, and adverse drug reactions. The long‐term goal of therapy includes maintaining quality of life and prevention of late complications related to atherosclerosis, progression of chronic renal failure, and death.

The mainstay treatment for nephrotic syndrome is corticosteroid therapy. Corticosteroids were found to be effective in inducing diuresis with loss of edema and reducing proteinuria in nephrotic syndrome [15]. As a result, the mortality rate among patients with childhood nephrotic syndrome has reduced to around 3%, with infection remaining the most common cause of death [16]. 75%–80% of SSNS children will experience one or more relapses, with 50% of these children having frequent relapses or steroid dependence [7–10]. Current KDIGO and IPNA guidance recommends oral prednisolone for relapse as a single daily dose of 60 mg/m^2^/day or 2 mg/kg/day, calculated using estimated dry weight and capped at 60 mg/day, until complete remission is maintained for at least three consecutive days. This should be followed by 40 mg/m^2^ or 1.5 mg/kg (maximum 40 mg) as a single alternate‐day dose for four weeks, without tapering [1, 7]. The term relapse treatment is more appropriate than induction therapy in this setting. It is noted that older guidelines such as those published by the American Academy of Pediatrics and the Indian Pediatric Nephrology Group had slightly different dosing regimens for prednisolone [8, 17]. The use of BSA over body weight in the dosing of prednisolone may reduce underdosing, although either approach may be used when based on estimated dry weight [7, 18]. Children receiving prednisolone at a dose of 2 mg/kg received only 85% of the 60 mg/m^2^ BSA dose, resulting in potential underdosing treatment [19]. An earlier study had demonstrated that when prednisolone was prescribed according to body weight, the relative underdosing percentage was significantly higher in frequent relapsers (16.6 ± 7.9%) compared to infrequent relapsers (8.7 ± 9.8%) (*p* = 0.03). Underdosing does not affect the initial response to treatment, but it increases the likelihood of a frequently relapsing course in responders [20].

The patient was given oral prednisolone 40 mg OD upon admission up to Day 3. His height was 138 cm, and his lowest recorded weight was 35.9 kg, giving a BSA of 1.17 m^2^ using the Mosteller formula. At 60 mg/m^2^/day, the uncapped calculated dose was approximately 70.4 mg/day, while 2 mg/kg/day would calculate to 71.8 mg/day; however, the guideline maximum is 60 mg/day [1, 7, 12]. Therefore, the revised total dose of 60 mg/day was the appropriate capped dose and was not underdosing. In contrast, 40 mg/day corresponded to approximately 34.1 mg/m^2^/day and was below the recommended relapse dose. The prescribed 30 mg twice‐daily regimen delivered the correct total daily dose, although current guidance prefers 60 mg as a single daily dose. Available evidence suggests that divided and single dosing have similar efficacy, but single dosing is preferred to simplify administration and support adherence [1, 7]. This increment in dosage was important because underdosing of prednisolone in nephrotic syndrome may contribute to delayed remission and prolonged exposure to complications associated with active nephrotic syndrome.

FRNS and SDNS are distinct relapsing patterns within SSNS. FRNS depends on relapse frequency within defined periods, whereas SDNS is characterized by relapse during recommended prednisolone treatment or within 14 days after discontinuation [7]. The patient in this report had SDNS, and the need for long‐term glucocorticoid‐sparing therapy was consistent with his steroid‐dependent disease course. In cases of confirmed FRNS, SDNS, or steroid toxicity, guidelines recommend corticosteroid‐sparing agents rather than no treatment or continuation of corticosteroid alone [1, 7, 8, 10, 12, 17, 21]. The recommended agents in corticosteroid‐sparing regimens include cyclophosphamide, ciclosporin, mycophenolate mofetil, levamisole, and rituximab. A retrospective study assessing the safety and efficacy of corticosteroid‐sparing agents in FRNS/SDNS reported that levamisole, mycophenolate mofetil, cyclophosphamide, and ciclosporin significantly reduced the relapse rate and cumulative steroid dose (*p* < 0.0001). The treatment success rate was highest with ciclosporin (69.2%) but was not statistically significant when compared to the other agents, and no serious adverse effects were observed with any of the four agents during the study period [22].

Specifically for levamisole, a recent observational study on 81 children with FR/SDNS aimed at determining the effectiveness of levamisole in maintaining remission reported that levamisole was effective in maintaining remission in 90% of children while on treatment as compared to 76.4% following drug discontinuation. Furthermore, levamisole was also found to significantly reduce the episodes of relapses as well as cumulative prednisolone doses [23, 24]. Following these findings, some guidelines recommend levamisole as the first‐line steroid‐sparing agent before offering alternative corticosteroid‐sparing therapy in children with FR/SDNS.

The efficacy of rituximab as an alternative for corticosteroid‐sparing therapy in children with FR/SDNS has also been proven. A multicenter, double‐blind, randomized, placebo‐controlled trial that investigated the efficacy and safety of rituximab in patients with high disease activity reported significantly longer median relapse‐free period in the group receiving rituximab (267 days, 95% CI: 223–374) compared to those receiving placebo (101 days, 70–155; hazard ratio: 0·27, 0·14‐0·53; *p* < 0.0001) [25]. In a very recent systematic review and meta‐analysis, rituximab significantly reduced the rate of relapses (OR = 0.11; 95% CI: 0.03, 0.43; *p* = 0.001), rate of steroid or/and calcineurin inhibitors use (OR = 0.05; 95% CI: 0.01, 0.28; *p* = 0.0007), and the dosage of steroid used compared to those in the control group (standardized mean difference [SMD] = −1.49; 95% CI: −2.00, −0.99; *p* < 0.00001) [26].

For ciclosporin and mycophenolate mofetil, comparison between the two agents showed ciclosporin to be more superior in preventing relapses in children with FRNS and inducing complete remission in children with SRNS [27]. These findings confirmed an earlier randomized prospective crossover trial, which reported that 36% of children receiving mycophenolate mofetil therapy experience relapse compared to only 15% among those receiving ciclosporin (*p* = 0.06). Furthermore, during the first year, time without relapse was significantly longer among those who received ciclosporin as compared to mycophenolate mofetil (*p* < 0.05), but not during the second year (*p* = 0.36) [28]. When comparing mycophenolate mofetil to levamisole, a single‐center, randomized, open‐label trial which enrolled 149 children ages 6–18 years old with FR/SDNS reported that therapy with mycophenolate mofetil was not superior to levamisole in terms of the proportions of participants with sustained remission (40.8% vs. 34.2%), frequent relapses (14.5% vs. 16.4%), or treatment failure, a composite outcome of frequent relapses, steroid resistance, or significant steroid toxicity (15.8% vs. 20.6) [29].

It is well established that calcineurin inhibitors such as ciclosporin may lead to adverse effects such as neurotoxicity, hypertension, hirsutism, gingival hyperplasia, and nephrotoxicity [30]. In this patient, ciclosporin was selected for SDNS after relapse despite a previous course of cyclophosphamide and the development of glucocorticoid toxicity. The maintenance dose of 50 mg twice daily (100 mg/day; approximately 2.8 mg/kg/day) was below the usual starting dose. The 12 h trough concentration of 128 ng/mL was within the KDIGO target of 60–150 ng/mL and the local laboratory range, although it exceeded the narrower IPNA target of 60–100 ng/mL; thus, subsequent dosing should continue to aim for the lowest exposure that maintains remission [1, 7]. Long‐term ciclosporin therapy should include regular blood pressure, renal function, electrolyte, and therapeutic drug monitoring. Kidney biopsy performed in January 2021 showed minor glomerular change, a minimal‐change pattern compatible with the patient’s steroid‐sensitive phenotype. The principal concern was therefore not the immediate indication or measured exposure, but the five‐year duration of therapy. IPNA recommends avoiding prolonged CNI use beyond a total of 2–3 years where possible, while KDIGO advises considering kidney biopsy after prolonged exposure to assess for nephrotoxicity [1, 7]. The 2021 biopsy predates much of the patient’s subsequent ciclosporin exposure and therefore cannot exclude later chronic CNI nephrotoxicity. Continued therapy should be actively reassessed by pediatric nephrology, using the lowest effective dose with serial blood pressure, kidney function, electrolytes, and trough levels; repeat biopsy may be considered if kidney function declines or prolonged continuation remains necessary. Cosmetic‐associated adverse effects of ciclosporin should also be recognized, as it may be of importance to a growing teenager. Alternative glucocorticoid‐sparing therapy may be considered according to relapse control, previous treatment response, adverse effects, cost, and patient preference [31].

### 2.2. Management of Complications of Nephrotic Syndrome–Edema

Edema is one of the most common presentations of nephrotic syndrome due to the loss of albumin in the urine and fluid shift from intravascular to the interstitial compartment. The immediate goals of therapy are to relieve signs and symptoms of fluid overload, to reduce body weight by 1% per day, and to minimize ascitic fluid volume and decrease peripheral edema, without causing intravascular volume depletion. The long‐term goal of therapy is to maintain the quality of life among children with nephrotic syndrome.

Since children with nephrotic syndrome are at risk of intravascular volume depletion, diuretics are generally not necessary, but they may be used with caution if required. Assessment of intravascular volume, blood pressure, urine output, serum electrolytes, and kidney function should precede diuretic therapy. In symptomatic grossly edematous states, human albumin (20%–25%) at a dose of 0.5–1.0 g/kg can be given in combination with IV frusemide 1–2 mg/kg to produce diuresis in selected patients, particularly when there is severe edema with suspected intravascular depletion or inadequate response to cautious loop‐diuretic therapy [7, 12]. Frusemide is the principal diuretic with a dose of 1–3 mg/kg OD for three to five days. Other classes of diuretics such as thiazide diuretics and potassium‐sparing diuretics can be added if there is inadequate response to loop diuretics and renal function and electrolytes permit. In diuretic‐resistant edema, IV human albumin 1 g/kg is recommended along with IV frusemide 2–3 mg/kg [32]. The co‐administration of human albumin with frusemide resulted in greater median urine volume excretion (*p* = 0.01), greater median daily sodium excretion (*p* = 0.08), and more weight loss (*p* = 0.006) compared to frusemide alone [33]. Nevertheless, albumin‐assisted diuresis should not be presented as universal first‐line therapy because it should be reserved for carefully selected and monitored patients.

Despite being admitted due to gross edema, the described patient was initially given oral spironolactone 25 mg BD monotherapy on Day 4 of admission. Serum electrolytes and renal function had been reviewed, and serum potassium had decreased from 5.4 to 4.2 mmol/L before spironolactone was initiated; therefore, it was not started during active hyperkalemia. IV Human albumin and frusemide were only administered on Day 6 of admission after worsening edema and a marked increase in body weight. We suggest against administering spironolactone as initial monotherapy for gross edema because aldosterone antagonists such as spironolactone have a weak diuretic effect. They are generally used in combination with loop or thiazide diuretics to prevent hypokalemia and provide a synergistic diuretic effect [34]. Additional treatment with spironolactone should only be given to children who require higher doses and prolonged treatment with frusemide and after serum potassium and renal function have been checked [7, 9]. In this patient, the earlier hyperkalemia had resolved before spironolactone was commenced; nevertheless, ongoing potassium and renal‐function monitoring remained necessary. In children with severe or symptomatic edema, especially after assessment of intravascular volume status, cautious use of loop diuretics with IV albumin reserved for selected patients should be considered. Spironolactone should not be used as initial monotherapy for gross edema, particularly in the presence of ongoing hyperkalemia.

### 2.3. Management of Complications of Nephrotic Syndrome–Infection Prophylaxis

Due to the damaged glomerulus, complement components and immunoglobulins are lost in the urine, which results in an increased risk of infection in children with nephrotic syndrome. Earlier studies have shown that infections such as spontaneous bacterial peritonitis and cellulitis are common among children with nephrotic syndrome. Intercurrent infections, particularly upper respiratory infections, are also common triggers of relapse. In this patient, the respiratory symptoms were temporally associated with relapse, although neutrophilic leukocytosis alone did not confirm a bacterial infection. The immediate goals of therapy for the management of infection related to nephrotic syndrome are to identify and eradicate an underlying pathogen when present and to prevent the development of sepsis or septic shock. Indeed, sepsis remains one of the main causes of death among children with nephrotic syndrome. The long‐term goals of therapy are to prevent immediate complications such as thromboembolic events, infection, and drug reactions apart from maintaining quality of life.

*Streptococcus pneumoniae* is the most common organism in primary peritonitis apart from other organisms such as β‐hemolytic streptococci, *Haemophilus*, and Gram‐negative bacteria [35]. There were mixed recommendations among the available guidelines regarding the use of antibiotic prophylaxis for children with nephrotic syndrome. Some guidelines recommend the administration of penicillin V 125 mg BD for 1–5 years old, 250 mg BD for 6–12 years old, and 500 mg BD for those more than 12 years old at diagnosis and during relapses, especially in patients who presented with gross edema [12]. However, due to lack of evidence to support the use of prophylactic penicillin in peritonitis, the American Academy of Pediatrics does not support routine administration of antibiotic prophylaxis during relapse or remission of nephrotic syndrome [17]. The rationale behind the use of penicillin as antibiotic prophylaxis against peritonitis was extrapolated partly from studies in children with sickle cell disease [36]. To date, no randomized controlled trials on antibiotic prophylaxis have been performed in children with nephrotic syndrome. Therefore, penicillin V in this case should be described as a local, individualized prophylactic decision rather than a universal standard. The subsequent increase to four‐times‐daily dosing represented empirical treatment‐intensity dosing, although a definite bacterial focus was not documented. Despite recommendations to use antibiotic prophylaxis for younger patients with persistent anasarca, SRNS, or FRNS, the risk of developing drug resistance should be balanced against the limited evidence supporting this practice. The Italian Society for Pediatric Nephrology recommends against the routine use of antibiotic prophylaxis for children with nephrotic syndrome [9].

### 2.4. Management During Intercurrent Infection

The patient remained hemodynamically stable and was able to take oral medication. Continuation of ciclosporin was therefore clinically defensible in the absence of documented severe sepsis, acute kidney injury, or calcineurin‐inhibitor toxicity; temporary interruption should be considered if infection is severe, kidney function deteriorates, oral intake is unreliable, or an interacting antimicrobial is required. This decision to continue ciclosporin during the acute admission should be distinguished from the separate need to reassess its long‐term use after five years of exposure. Although prolonged glucocorticoid exposure can create a need for stress‐dose coverage during illness, prednisolone 60 mg/day was already above usual stress‐dose equivalence. Additional parenteral hydrocortisone would generally be reserved for hemodynamic instability, suspected adrenal crisis, persistent vomiting or diarrhea, or inability to take or absorb oral glucocorticoids [14].

### 2.5. Clinical Recommendations

This case report describes a relapse of nephrotic syndrome despite ciclosporin therapy in a child with SDNS, successfully managed with correction of the oral prednisolone relapse dose and continued ciclosporin. The goals of therapy are to achieve remission promptly and maintain remission while minimizing treatment toxicity. Corticosteroid therapy remains the mainstay treatment for nephrotic syndrome but carries adverse effects, especially among steroid‐dependent and frequently relapsing patients receiving repeated or long‐term therapy. Corticosteroid‐sparing regimens therefore play a vital role. Unfortunately, there is no common consensus that one agent should always be used before another because comparative evidence remains limited. Levamisole may be considered a suitable steroid‐sparing option where cost, tolerability, and safety are important considerations. However, the choice among levamisole, cyclophosphamide, mycophenolate mofetil, calcineurin inhibitors, and rituximab should be individualized according to disease pattern, previous response, adverse‐effect profile, adherence, availability, cost, and patient preference. For this patient, five years of ciclosporin exposure warrants active pediatric‐nephrology reassessment of the continuing indication, the lowest effective dose, alternative glucocorticoid‐sparing options, and whether further evaluation for chronic CNI toxicity is required; a therapeutic trough concentration alone does not exclude cumulative nephrotoxicity.

Diuretic therapy is also an important component in the management of nephrotic syndrome, especially among those with signs and symptoms of clinically important edema or fluid overload. The selection of diuretic agent is crucial to ensure adequate diuresis and prevent hypovolemia. Cautious loop‐diuretic therapy is generally preferred for symptomatic edema; IV human albumin in combination with frusemide should be reserved for selected patients with severe or refractory edema or suspected intravascular depletion rather than recommended as universal first‐line therapy. Owing to its poorer efficacy and potassium‐sparing effect, spironolactone should not be used as first‐line monotherapy for gross edema, particularly before electrolytes and renal function have been assessed. Despite the risk of infection caused by *Streptococcus pneumoniae*, routine antibiotic prophylaxis should not be recommended for all children with nephrotic syndrome. Instead, prophylaxis should be considered case by case after weighing infection risk, degree of edema, vaccination status, local practice, antimicrobial resistance, and the limited direct evidence.

## 3. Conclusion

This case highlights the practical challenges of managing a relapse in a child with SDNS receiving long‐term ciclosporin therapy. The relapse was successfully managed by correcting prednisolone to the recommended capped dose, together with appropriate supportive treatment for edema and infection risk. Continued follow‐up should include regular assessment of disease control, renal function, blood pressure, ciclosporin exposure, and treatment‐related adverse effects, with the glucocorticoid‐sparing regimen reviewed according to clinical response and tolerability.

## Author Contributions

All authors contributed to the conception and clinical interpretation of the case, reviewed the literature, and drafted or critically revised the manuscript.

## Funding

The authors received no financial support for the research, authorship, and/or publication of this article.

## Disclosure

All authors meet the ICMJE authorship criteria. All authors approved the final version and agree to be accountable for the work.

## Consent

Written informed consent was obtained from the patient’s parent or legal guardian for his anonymized information to be published in this article.

## Conflicts of Interest

The authors declare no conflicts of interest.

## Data Availability

Data sharing is not applicable to this article as no datasets were generated or analyzed during the present study.
